# Generation of Neural Progenitor Cells From Canine Induced Pluripotent Stem Cells and Preliminary Safety Test in Dogs With Spontaneous Spinal Cord Injuries

**DOI:** 10.3389/fvets.2020.575938

**Published:** 2020-11-05

**Authors:** Lyndah Chow, Stephanie McGrath, Camila de Arruda Saldanha, Lawrence R. Whalen, Rebecca Packer, Steven Dow

**Affiliations:** ^1^Department of Clinical Sciences, College of Veterinary Medicine and Biomedical Sciences, Center for Immune and Regenerative Medicine, Colorado State University, Ft. Collins, CO, United States; ^2^Department of Clinical Sciences, College of Veterinary Medicine and Biomedical Sciences, Colorado State University, Ft. Collins, CO, United States; ^3^Department of Biomedical Sciences, College of Veterinary Medicine and Biomedical Sciences, Colorado State University, Ft. Collins, CO, United States

**Keywords:** neural progenitor, induced pluripotent stem cell, spinal cord injury, spinal cord injection, teratoma

## Abstract

Advances in stem cell technology, including the use of induced pluripotent stem cells (iPSC) to produce neurons and glial cells, offer new hope for patients with neurological disease and injuries. Pet dogs with spinal cord injuries provide an important spontaneous animal model for evaluating new approaches to stem cell therapy. Therefore, studies were conducted to identify optimal conditions for generating neural progenitor cells (NPC) from canine induced pluripotent stem cells (iPSC) for preliminary evaluation in animals with spinal cord injury. We found that canine NPC could be induced to differentiate into mature neural cells, including glia and neurons. In addition, canine NPC did not form teratomas when injected in NOD/SCID mice. In a pilot study, two dogs with chronic spinal cord injury underwent fluoroscopically guided intrathecal injections of canine NPC. In follow-up MRI evaluations, tumor formation was not observed at the injection sites. However, none of the animals experienced meaningful clinical or electrophysiological improvement following NPC injections. These studies provide evidence that canine iPSC can be used to generate NPC for evaluation in cellular therapy of chronic spinal cord injury in the dog spontaneous injury model. Further refinements in the cell implantation procedure are likely required to enhance stem cell treatment efficacy.

## Introduction

Stem cell therapy offers new hope for patients suffering from spinal cord injury (SCI) (1, 2). A number of small clinical trials have investigated the potential for stem cell transplantation in SCI, including mesenchymal stem cells and neural stem cells derived from embryonic stem cells and directly from spinal tissue (3–7). In general, human clinical trials of stem cell therapy in SCI have demonstrated modest improvements in spinal cord function following cellular therapy, but recovery is varied and the optimal cell type for transplantation is yet to be determined (8). Induced pluripotent stem cells (iPSC) represent one potential new source of cells for therapy of SCI (9). Human iPSCs have been shown to differentiate into functional neurons and glial cells after engraftment into injured rat and mouse spinal cords; and grow long axons that extend through the injury sites and form connections with existing neurons (9–13). However, there are also significant potential risks to the use of iPSC derived stem cells for cellular therapy, including in particular the risk of the tumor formation, particularly teratomas (14); combined with the difficulties of generating stable, clinical grade iPS derived neural cell lines (15). Thus, there is considerable interest in determining whether iPSC-derived cells are effective for SCI, and whether they can be administered without the risk of tumor formation.

Large animal testing in models other than rodent induced SCI are essential to fully assess the safety and clinical efficacy of stem cell therapy (16, 17). Pet dogs with spontaneous SCI, typically due to intervertebral disk rupture or automobile trauma, offer a valuable and realistic animal model for spinal cord injury research. Numerous research studies with implanted mesenchymal stem cells (MSC) have been performed in research dogs with induced SCI (18–21). In addition, several clinical trials have reported the results of MSC injections in spontaneous, chronic SCI in dogs, with some evidence of increased locomotion, and no adverse effects from the injections (22–25). To date, we are unaware of the evaluation of iPSC-derived stem cells in natural or induced SCI in dogs.

Therefore, in this report we evaluated whether canine iPSC could be induced to differentiate into NPCs and to form mature neural cells *in vitro*. In addition, the safety and tumorogenicity of canine NPCs was evaluated in mouse models. Finally, studies were also done to assess the safety and potential efficacy of NPCs following intra-spinal injection in dogs with naturally-occurring spinal cord injuries.

## Materials and Methods

### Generation of Canine Neural Progenitor Cells (NPCs or NPC)

A canine iPS cell line was generated as previously described (26) from skin biopsy fibroblast derived from a 6-year old male standard poodle To differentiate iPS cells into Neurospheres (neural progenitor cells), colonies were digested to single cell suspension and re-suspended in media containing DMEM/F12 (27) (Life Technologies Corp. Grand Island NY), Antibiotic-Antimycotic, B27 (Thermo Fisher Scientific Waltham, MA), 20 ng/mL Recombinant Human FGF-basic (Fibroblast Growth Factor-basic, bFGF), 20 ng/mL Recombinant Human EGF (Epidermal Growth Factor) (PeproTech, Rock Hill, NJ), and 2 μg/mL Heparin (Sigma-Aldrich, St. Louis MO). Dissociated cells were plated in 100 mm ultra-low attachment culture dishes (Corning Inc. Corning, NY) at a density of 4 × 105 cells/mL, on day 10 neurospheres were collected and used for injection or for further differentiation.

### Generation of Mature Neurons and Astrocytes

NPCs were collected and plated on round cover slips coated with 20 μg/mL laminin and poly-ornithine (Sigma-Aldrich, St. Louis MO) in a 24 well cell culture plate (Corning Inc. Corning, NY). Mature neurons were grown in neuronal growth media containing DMEM/F12, B27, N2, Glutamax, antibiotic-antimycotic solution, and NEAA (Thermo Fisher Scientific Waltham, MA). Astrocytes were grown with the addition of 1% fetal bovine serum (VWR Life Science, Radnor, PA). Mature neurons and astrocytes were detached using Accutase (Stemcell Technologies Inc. Vancouver, Canada) after 14 days and processed for PCR or stained using ICC as described below.

### Generation of Oligodendrocytes

NPCs were plated onto laminin/ poly-ornithine coated cover slips in oligodendrocyte differentiation base media, containing DMEM/F12, 50% Glucose, Glutamax, Sodium Pyruvate. N2, penicllin-streptomcin, and 20 ng/mL bFGF was added to oligo base media from day 0–4, 20 ng/mL bFGF and EGF was added from day 5–8, bFGF, EGF and also 10 ng/mL PDGF-AA (Platelet-Derived Growth Factor-AA) (PeproTech, Rock Hill, NJ) was added from day 9–12. After day 12, 30 ng/mL of T3 (3,3,5 triiodo-thryonine) (Sigma-Aldrich, St. Louis, MO) was added to oligodendrocyte differentiation base until the appearance of oligodendrocytes (~6–8 days later), which were collected for PCR or stained using ICC.

### Immunocytochemical (ICC) Evaluation of Differentiated Neurons and Astrocytes

ICC was performed according to previously published protocol (28). Primary antibodies used in these studies include: anti-vimentin (clone V9; Merck Millipore, Billerica, MA), anti-TUJ1 (beta III tubulin; clone TUJ1 Covance Inc. San Diego, CA), and anti-MAP2α (clone AP20; Merck Millipore, Billerica, MA). Corresponding rabbit and mouse irrelevant isotype antibodies were used at concentrations matching the primary antibodies (eBioscience Inc, San Diego CA). Secondary antibodies used were donkey anti mouse IgG or donkey anti rabbit IgG (Jackson ImmunoResearch Laboratories, Inc. West Grove, PA) conjugated to Cy3. Visualization of florescence staining was performed on Olympus IX83 spinning disk confocal microscope. Images were imported as Tiff files to Photoshop CC 2015, and adjusted with high definition resolution (HDR) toning. For each stain, HDR toning was applied to corresponding isotype control stains as well.

### Immunocytochemical Evaluation of NPC Cultures

NPC immunostaining was performed according to previously published protocols (26, 29). Primary antibodies used were anti-Nestin (clone 10C2; Merck Millipore, Billerica, MA), anti-TUJ1 (beta III tubulin; clone TUJ1 Covance Inc. San Diego, CA), anti-Vimentin (clone V9; Merck Millipore, Billerica, MA), anti-Oct3/4 (clone H134, Santa Cruz Biotechnology, Inc. Dallas, TX), anti-Nanog (Clone H-155 Santa Cruz Biotechnology Inc. Dallas, TX).

### Gene Expression Profiles of NPC and Differentiated Lineage Cells

Total mRNA was isolated from each cell type using the RNeasy kit (Qiagen, Germantown, MD). cDNA was synthesized using a QuantiTect Reverse Transcription Kit (Qiagen, Germantown, MD) according to the manufacture's protocol. Primers for RT-PCR were designed using Primer-BLAST (NCBI) (Table 1). RT-PCR reaction was performed using iQ SYBR Green Supermix (Bio Rad Hercules, CA). Amplification was performed by the MX3000P (Agilent Technologies, Inc. Santa Clara, CA). Data including amplification plots and dissociation curves was then analyzed using the Mx3000p version 2.0 software. Fold change was calculated using the ddCt method, Ct values were normalized to canine fibroblasts derived from the same donor dog as the iPSC, using the housekeeping gene GAPDH.

**Table 1:** Primers used for RT-PCR.

**Gene**	**Forward**	**Reverse**	**Gene accession number**
**NPC**
SOX2	AACCCCAAGATGCACAACTC	CGGGGCCGGTATTTATAATC	XM_005639752.3
Kit	GAGCTCAGAGTCTATCGCAGC	AGATGGTTGAGAAGAGCCTGTC	NM_001003181.1
NKX6.1	AACACACGAGACCCACGTTT	GGAACCAGACCTTGACCTGA	XM_544960.6
**Neuron**
NeuN (RBFOX3)	CCCTACAGTAACGGCTGGAA	TGATACACGACCGCTCCATA	XM_022422656.1
Map2a	GACATGCAAGGCACAGAAGA	AGGTGAGATGGGAGCAGCTA	XM_022415214.1
NeuroD6	CTGAGAATCGGCAAGAGACC	GCTGTGGTAGGGTGGGTAGA	XM_539504.6
**Astrocyte**
GFAP	GCAGATGAAGCCACCTTAGC	TCTTAGGGCTGCTGTGAGGT	XM_843285.5
NGF	AAGCTTCAGCATTCCCTTGA	TGCTCCTGTGAGTCCTGTTG	NM_001194950.1
RASSF10	CCGAGGGGTGGGAAGTT	GAGCCAGGGCAGGTGTC	XM_849324.4
**Oligodendrocyte**
OLIG2	CATTCGGTACCAGGAGCACT	CTGGCACTGTTGACAAAGGA	XM_005638837.3
OMG	TATGCACAGAGAGGCACAGG	CAGGGTCCTCAGATTGGTGT	XM_848798.5
MBP	GCAGATGTGGAGCAGAACAA	GTCCTCTTGGATGGTCTGGA	XM_005615298.3
**Housekeeping**
GAPDH	ATCACTGCCACCCAGAAGAC	TCAGCTCAGGGATGACCTTG	NM_001003142.2

### Preparation of NPC for Injection

NPC were collected from cell culture dishes by gravitational settling. Spheroids were then dissociated into single cell suspension using Accumax cell detachment solution (Stemcell Technologies Vancouver, BC). Cells were washed twice using sterile PBS, and resuspend at a concentration of 2 × 10^7^ /mL in sterile PBS.

### Enrollment and NPC Administration to Dogs With Spinal Cord Injury

This study was conducted in accordance with appropriate IACUC and Clinical Review Board protocols, permissions, or exemptions (CRB protocol number VCS #2015-016).

Three pet dogs with naturally-occurring SCI were evaluated at the Colorado State University Veterinary Teaching Hospital for management of chronic (>4 weeks duration) severe thoracolumbar spinal cord injury. These animals met study eligibility requirements and were enrolled in the stem cell trial after owners signed informed consent. Enrollment criteria included the following: focal or multifocal spinal cord injury caudal to T3 (based on neurological exam and MRI), absence of perceptible nociception, and lack of voluntary ambulation using the pelvic limbs for at least 1-month post injury. One dog was excluded from the study due to Unable to undergo anesthesia for the subsequent MRI and electrodiagnostic testing due to grave comorbidities and was euthanized for unrelated reasons.

Stem cell injections were done in the T3-S1 spinal cord segments, with the exact locations determined by MRI images of the spinal cord lesions. Fluoroscopy was used to guide a 22-gauge spinal needle through the interarcuate ligament to the ventral spinal canal. The NPC were injected into 3 sites in each dog: the center of the spinal lesion, the spinal cord one vertebral space cranial to the lesion, and into the spinal cord one vertebral space caudal to the primary lesion site. Each injection site received 2 × 10^6^ NPC., in a total injection volume per site of 100 ul. Each dog was maintained on daily cyclosporine therapy at a 5 mg/kg BID for 24 weeks to help prevent rejection of engrafted NPC.

### MRI Imaging

Two dogs enrolled in the study had total of three T3-S3 MRI studies performed. A pre-treatment MRI was performed immediately prior to NPC injection. The initial MRI study included the following sequences: T1- and T2-weighted sagittal images, T1- and T2-weighted transverse images, and DTI images.

### Electrodiagnostic Studies

Evoked spinal cord and somato-sensensory potential studies were performed in each dog, under general anesthesia (30). For the evoked spinal cord potentials, anodal stimulating electrodes were placed at the level of the greater trochanter of the femur and just proximal to the tarsus. Corresponding cathodal electrodes were placed 5–7 mm proximal to the anodes. Electrical pulses were of 0.05 ms in duration and delivered at a rate of 4 pulses/second. Evoked potentials were recorded at each interarcuate space between the 7th lumbar vertebra and two spaces cranial to the site of the lesion. An active recording electrode with a 2 mm bared tip was placed percutaneously into each interarcuate ligament. A reference electrode with a 3 mm bared tip was placed in the epaxial muscles 1–2 cm lateral to the active electrode. A ground electrode was placed subcutaneously within 5 cm of the recording site. The stimulations were repeated in exactly the same fashion from both pelvic limbs. Simultaneous with the other recordings, somatosensory evoked potentials were recorded percutaneously from the skull overlying the somatosensory cortex contralateral to the stimulated nerve. The active recording electrode was placed against the bone overlying the somatosensory cortex with the reference electrode placed subcutaneously at the intercanthal space.

## Results

### NPC Induction From Canine iPSC

**Figure 1:**
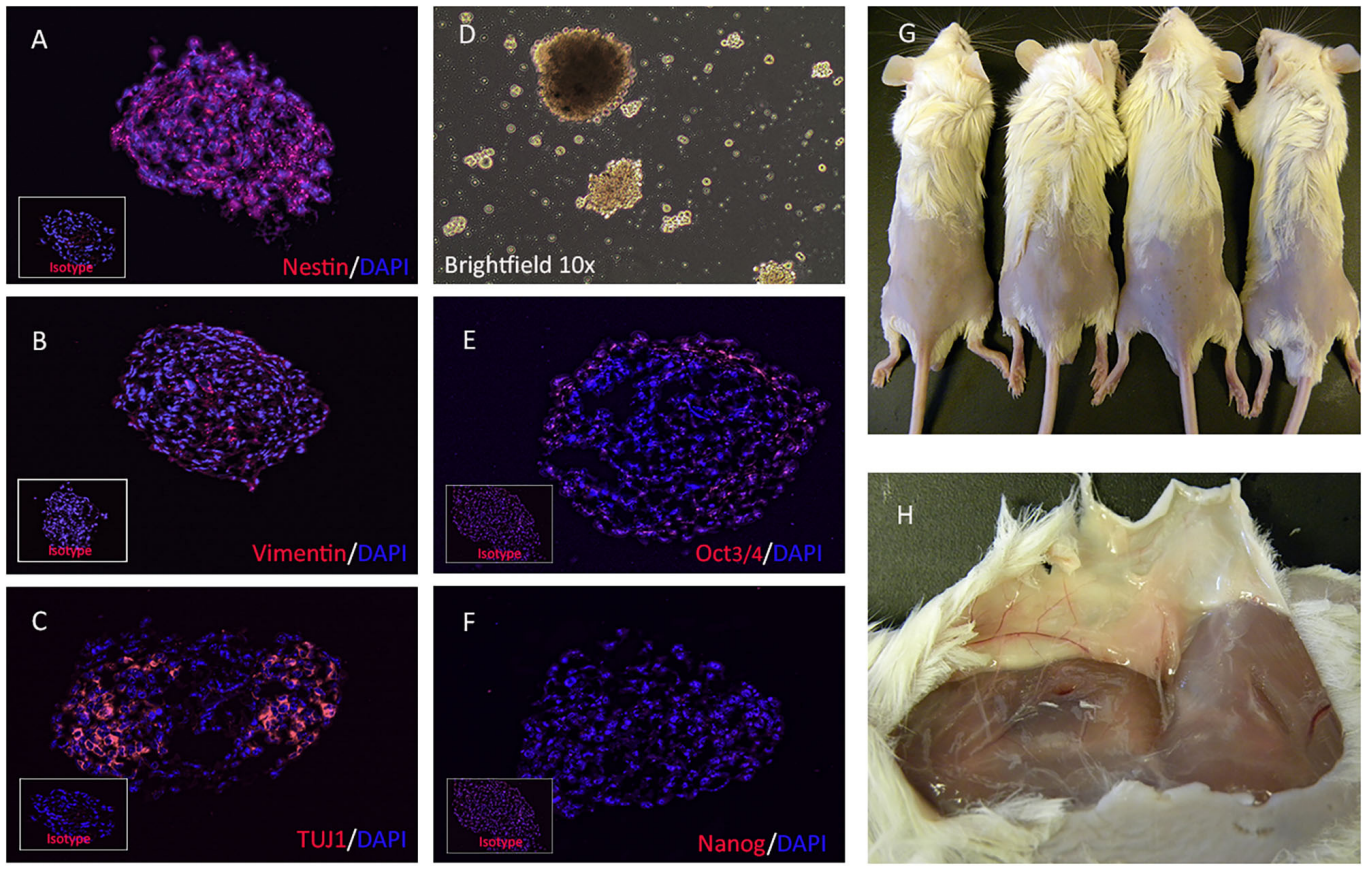
Immunofluorescence characterization and teratogenicity of NPC. NPCs immunostained for detection of intracellular neural stem cell specific antigens, including nestin **(A)**, vimentin **(B)**, and beta-tubulin **(C)**, and pluripotency factors Oct3/4 **(E)** and Nanog **(F)**. Bright field image showing cluster of neural stem cells formed *in vitro* **(D)**. Inset boxes depict staining with corresponding isotype antibodies. Dissociated NPCs were embedded in Matrigel and injected s.c in NOD/SCID mice. Teratomas failed to form in any of the 4 mice injected with NPCs **(G)** and tumor tissues was also not detected after subcutaneous dissection **(H)**.

Cellular aggregation was first observed 24 h after dissociation of iPSC and placement in neurosphere medium, After 10 days, dissociated iPSC cells formed spheroids consisting of >50 cells (Figure 1D). These NPCs expressed the neural stem cell marker nestin (Figure 1A) (31), the intermediate filament protein vimentin (Figure 1B), and the neuron-specific class III β-tubulin (TUJ1) protein (Figure 1C), as revealed by immunocytology. There was low expression of pluripotency marker Oct3/4 (POU5F1) (Figure 1E) and no expression of stem cell factor Nanog (Figure 1F). NPC were also negative for astrocyte marker GFAP (Glial fibrillary acidic protein) and post mitotic neuronal marker MAP2a (Microtubule-Associated Protein 2).

### Evaluation of NPC Cells for Teratoma Formation in Immune Deficient Mice

To address this possibility of teratoma formation from canine NPC cells, NOD/SCID Mice (*n* = 4 per group) were injected s,c with 2 × 10^6^ NPC embedded in Matrigel. Mice were observed for 90 days for s.c. tumor formation. Tumor formation was not observed at euthanasia 90 days post-injection (Figure 1G), and tumor tissue could not be detected in the dissected subcutaneous tissues in any of the 4 mice (Figure 1H).

### *In vitro* Differentiation of NPC to Neuronal and Glial Subtypes

**Figure 2:**
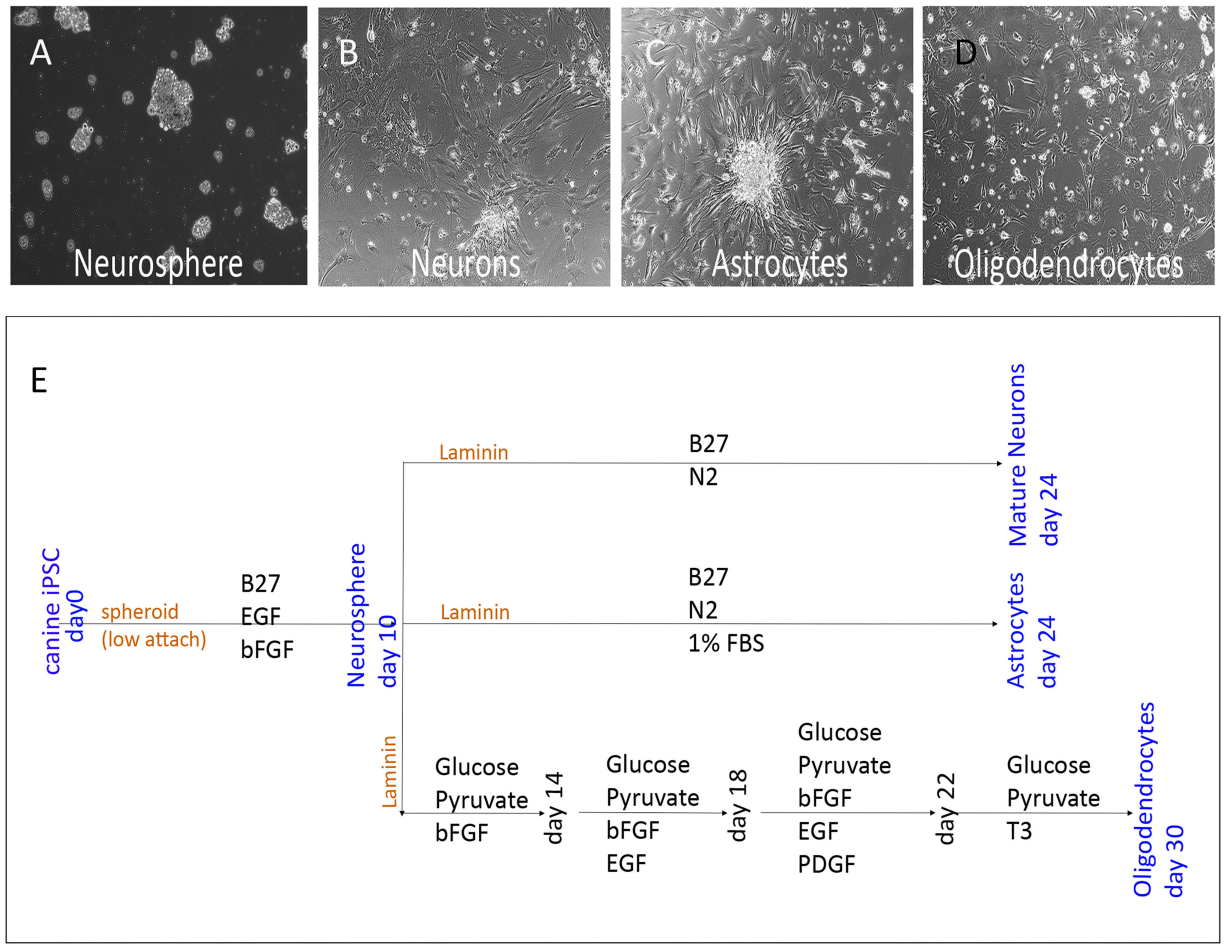
Lineage specific differentiation scheme of NPC. Bright field images (10x) of NPC cultures **(A)** showing spheroidal clusters in suspension, as well as differentiated neurons **(C)** with elongated thin projections adhered to culture dish. Astrocytes **(B)** with star shaped glial morphology, and oligodendrocytes **(D)** with branching microglia. Schematic of the cell culture differentiation protocol, including growth factors and substrates used as well as differentiation time **(E)**.

**Figure 3:**
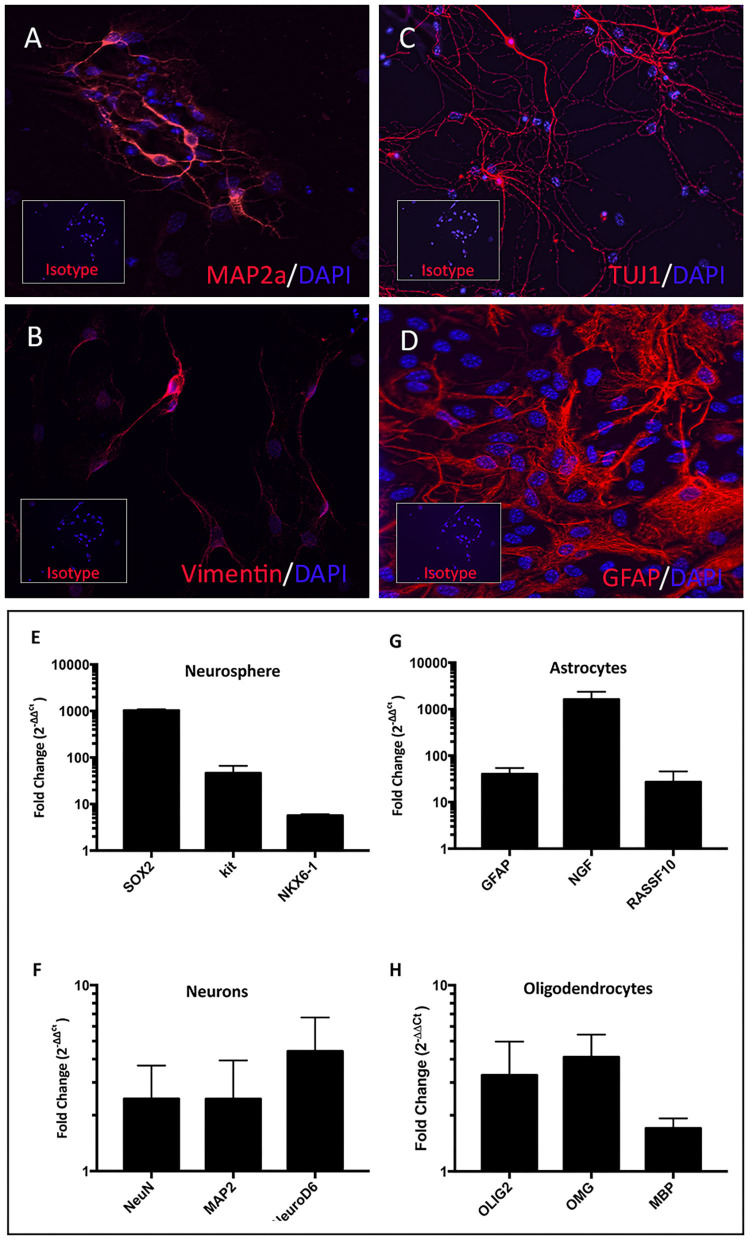
Characterization of terminally differentiated neural cells. Differentiated neurons expressed lineage specific markers MAP2a **(A)**, vimentin **(B)**, and TUJ1 **(C)**. Astrocytes expressed GFAP **(D)**. Inset boxes depict staining with appropriate isotype control antibodies. NPC cultures exhibited upregulated SOX2, Kit, and NXK6.1 compared to skin fibroblasts derived from the same animal **(E)**. Fold changes shown in log scale on y-axis. Astrocyte cultures upregulated mRNA for GFAP, NGF, and RASSF10 **(G)**. Mature neuron cultures upregulated NeuN, MAP2, and NeuroD6 **(F)**, while oligodendrocyte cultures upregulated OLIG2, OMG, and MBP **(H)**.

To verify that NPC cultures contained multipotent progenitor cells, NPC were plated in differentiation media (32) (Figure 2E). The NPC (Figure 2A) differentiated into astrocytes (Figure 2B), mature neurons (Figure 2C), and oligodendrocytes (Figure 2D). The NPC derived neurons expressed MAP2a (Microtubule-Associated Protein 2), a neuron specific protein found in post-mitotic neurons (Figure 3A) (33). In addition, differentiated neurons also expressed TUJ1 (neuron-specific class III β-tubulin), a marker found in immature neurons (Figure 3C), and type III intermediate filament protein Vimentin, which is found in growing axonal neuritis (34) (Figure 3B). NPC cultures could also be induced to differentiate into astrocytes with the addition of 1% FBS, as revealed by expression of the astrocyte marker GFAP (Figure 3D) (35). Astrocyte cultures were negative for neuronal markers MAP2a and TUJ1 (data not shown). Differentiated cells were also evaluated for expression of stem cell, neuronal, astroglial, or oligodendroglial genes, using qRT-PCR. NPC cultures were found to upregulate expression of mRNA for SOX2 (SRY-box 2), stem cell factor receptor kit, and the transcription factor NKX6.1 (NK6 Homeobox 1) (Figure 3E) (27). Neuronally-differentiated cells upregulated expression of the nuclear protein RNA binding protein, and neuronal differentiation antigen 6 (NeuroD6) (36), while astrocytic cultures exhibited upregulated expression of mRNA for nerve growth factor and Ras Association Domain Family Member 10 (RASSF10) (Figure 3G) (37). Oligodendrocyte cultures had upregulated expression of oligodendrocyte lineage transcription factor 2 (OLIG2), oligodendrocyte myelin glycoprotein (OMG), and myelin basic protein (MBP) (Figure 3H) (38).

### Evaluation of Spinal Cord Injection Sites Following Intraspinal Injection of NPC

**Figure 4:**
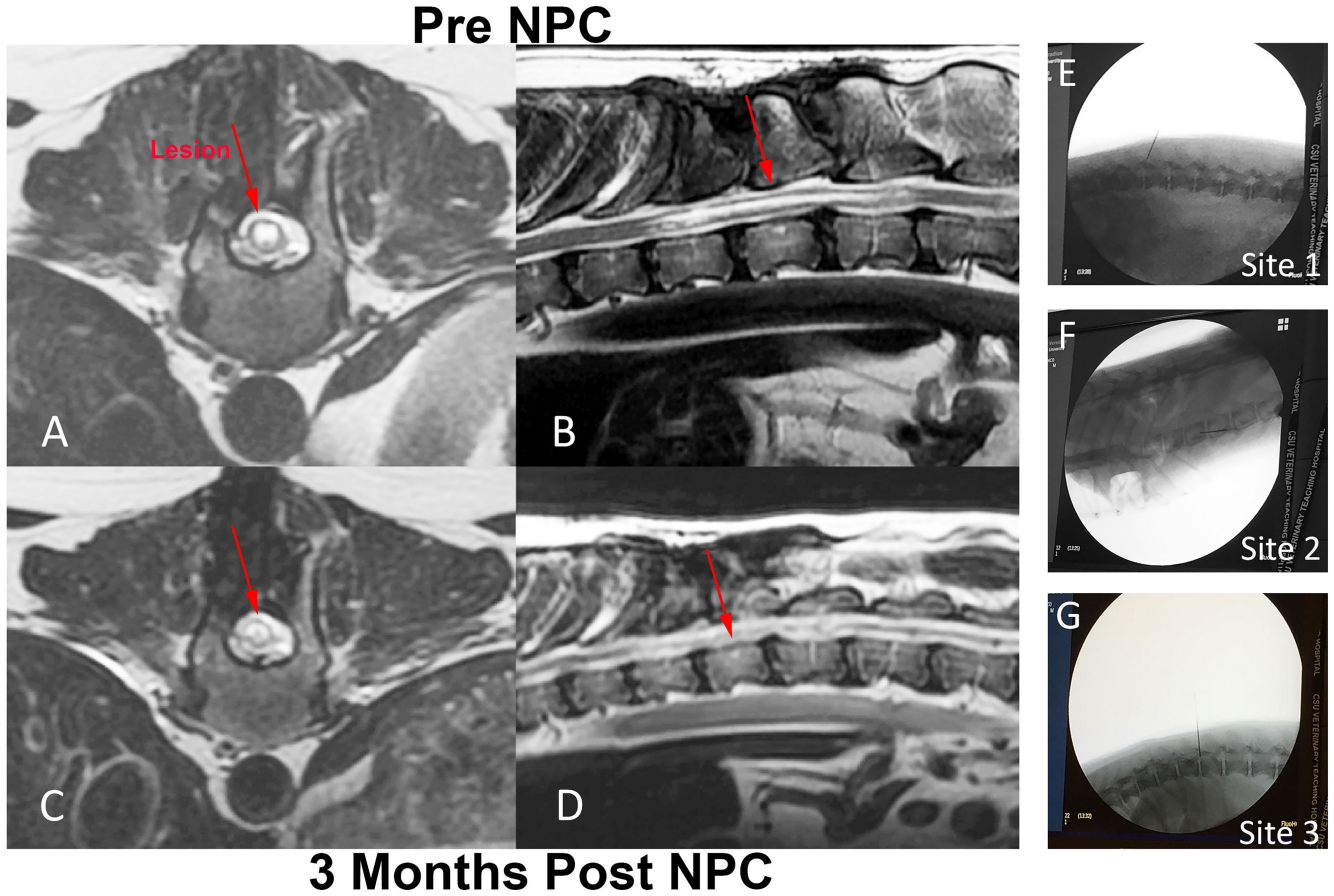
Intra-spinal NPC injection procedure and MRI evaluation of injection sites in a dog with chronic SCI. Fluoroscopic images of injection sites including original injury site **(E)**, as well as an injection location cranial **(F)** and caudal **(G)** to the original injury site. T2-weighted MR images were obtained from dog 2, immediately prior to NPC injection **(A,B)** and 3 months after the intra-spinal injection procedure **(C,D)**. Prior to NPC injection, there was a T2 hyper-intense lesion extending from T11 to T13 present within the spinal cord on both the transverse and sagittal images (red arrows **A,B**). Following the NPC injections, the lesion at 3 months did not change in size or extent compared to the pre-injection images, nor was there evidence of tumor growth at the injection site (red arrows **C,D**).

Two dogs were included in the initial pilot study of NPC injection for SCI. NPCs were injected into 3 sites as described in materials and methods (Figures 4E–G). The spinal cord injuries were due to spontaneous, traumatic thoracolumbar disk rupture. Dog 1 had a spinal cord lesion at T11-T12, dog 2 had a spinal cord injury at T13-L1 (Figures 4A,C). Both dogs had undergone heminlaminectomies to decompress spinal cord compression at 2 months prior to study entry. Both dogs were evaluated clinically and by MRI examination following NPC administration at 3 and 6 months, with dog 2 also undergoing a third MRI examination at 12 months after NPC injection. At the time repeat MRI studies were done, neurological and electrophysiological evaluation was also performed. Neither of the animals experienced clinical or electrophysiological improvement following NPC injections; evoked potentials in control regions were consistent with normal reference ranges (30), and there were no evoked potentials when testing the injured regions both pre and up to 6 months post NPC injections. No notable changes were seen in the soft tissues or bony tissues of the spinal cord at either of the 3 injection sites (Figures 4B,D).

Following the NPC injections, the lesion at 3 months did not change in size or extent compared to the pre-injection images (red arrows Figures 4C,D). Glial scaring remains unchanged in intensity and extent before and after injections. In addition, there was no evidence of tumor formation at any injection site from the MRI evaluations.

In all treated dogs, no adverse effects that could be attributed to NPC injection were observed at any time point after NPC injection during the 6–12 month follow-up period. Clinical neurological evaluation including assessment of spontaneous ambulation and responses to cutaneous stimuli did not reveal any notable improvement in neurological function at any of the time points.

## Discussion

The implementation of iPSC-derived neural stem cells in human SCI trials requires first rigorous proof of their safety and efficacy using appropriate animal models. Dogs with naturally-occurring SCI offer a particularly compelling model of chronic SCI to model neural stem cell therapies for humans (16, 17, 39). This study is the first to our knowledge to show the differentiation of canine iPSC to NPC. The results of these characterizations were consistent with results in other species (15, 40), and also canine stem cell studies using embryonic or adipose stem cells (32, 41). We also demonstrate the safety of administering iPSC-derived NPC in dogs with follow-up for 6–12 months, particularly with respect to tumor formation at the injection site.

The other endpoint of the study was to evaluate the impact of NPC administration on recovery of neurological function in dogs with chronic SCI. Though this was a small pilot study involving only 2 animals, these early results suggest that direct intraspinal injection of cells alone is unlikely to provide significant functional improvement. There are several potential explanations for the failure to observe clinical benefit. For one, direct cell injection without surgical debridement of the spinal injury site does not remove debris and scar tissue from the original injury, which likely significantly impairs any regeneration from the injected NPC. Indeed, others have reported in non-human primate models that surgical access to the injection site is essential to provide a receptive bed for stem cell administration (42). It is also reported that stem cells must be administered in a matrix such as collagen or fibrin matrix (43) to retain the cells in the lesion site and to promote survival and axonal connection. In addition, many experimental stem cell treatments for SCI have been evaluated in animals within weeks after a spinal cord lesion was administered, at a time when significant gliosis and scarring had not occurred (8). In contrast, all 3 animals in the present study had SCI of at least 60 days duration, at time frame where spontaneous recovery would be considered a remote possibility. These important differences between acute and chronic SCI also play an important role in regulating the different responses to stem cell injections and survival (44).

Considering our findings from this pilot study, it will be interesting in the future to continue to evaluate stem cell therapies in the dog chronic SCI model, which provides a much more realistic animal model for evaluation of new cellular therapies for SCI treatment in humans than current rodent models. The availability of canine iPSC, and the technologies to generate large numbers of NPC should also facilitate these new studies.

## Conclusion

In this study, we have shown for that canine iPSC are able to differentiate into neuronal and glial lineage cells following the induction of NPC. Differentiated cells were verified by ICC and PCR. A total of two dogs were treated with iPS derived NPC for chronic spinal cord injury up to 4 months post injury. We concluded that to increase the chance of improvement in a chronic injury such as these, additional steps must be taken prior to the intra-spinal injection of cells. These steps may include surgical debridement of the original injury site, embedding of the NPC in a matrix, and also possibly an earlier administration of NPC within weeks of injury.

## Data Availability Statement

The raw data supporting the conclusions of this article will be made available by the authors, without undue reservation upon reasonable request.

## Ethics Statement

All procedures involving live animals were approved by the Institutional Animal Care and Use Committee at Colorado State University. Written informed consent was obtained from the owners for the participation of their animals in this study.

## Author Contributions

LC and SD wrote manuscript with support from SM. SM and RP performed neurological surgery. LW assisted with Fluoroscopy and MRI imaging. CA performed *in vitro* experiments. LC performed *in vitro* experiments and data analysis. All authors contributed to the article and approved the submitted version.

## Conflict of Interest

The authors declare that the research was conducted in the absence of any commercial or financial relationships that could be construed as a potential conflict of interest.
